# Building a Mobile HIV Prevention App for Men Who Have Sex With Men: An Iterative and Community-Driven Process

**DOI:** 10.2196/publichealth.4449

**Published:** 2015-11-16

**Authors:** Tamar Goldenberg, Sarah J McDougal, Patrick S Sullivan, Joanne D Stekler, Rob Stephenson

**Affiliations:** ^1^ School of Nursing Department of Health Behavior and Biological Sciences and the Center for Sexuality and Health Disparities University of Michigan Ann Arbor, MI United States; ^2^ Department of Medicine Division of Allergy and Infectious Diseases University of Washington Seattle, WA United States; ^3^ Rollins School of Public Health Department of Epidemiology Emory University Atlanta, GA United States

**Keywords:** HIV, AIDS, MSM, mobile app, prevention, community

## Abstract

**Background:**

Gay, bisexual, and other men who have sex with men (MSM) account for a disproportionate burden of new HIV infections in the United States. Mobile technology presents an opportunity for innovative interventions for HIV prevention. Some HIV prevention apps currently exist; however, it is challenging to encourage users to download these apps and use them regularly. An iterative research process that centers on the community’s needs and preferences may increase the uptake, adherence, and ultimate effectiveness of mobile apps for HIV prevention.

**Objective:**

The aim of this paper is to provide a case study to illustrate how an iterative community approach to a mobile HIV prevention app can lead to changes in app content to appropriately address the needs and the desires of the target community.

**Methods:**

In this three-phase study, we conducted focus group discussions (FGDs) with MSM and HIV testing counselors in Atlanta, Seattle, and US rural regions to learn preferences for building a mobile HIV prevention app. We used data from these groups to build a beta version of the app and theater tested it in additional FGDs. A thematic data analysis examined how this approach addressed preferences and concerns expressed by the participants.

**Results:**

There was an increased willingness to use the app during theater testing than during the first phase of FGDs. Many concerns that were identified in phase one (eg, disagreements about reminders for HIV testing, concerns about app privacy) were considered in building the beta version. Participants perceived these features as strengths during theater testing. However, some disagreements were still present, especially regarding the tone and language of the app.

**Conclusions:**

These findings highlight the benefits of using an interactive and community-driven process to collect data on app preferences when building a mobile HIV prevention app. Through this process, we learned how to be inclusive of the larger MSM population without marginalizing some app users. Though some issues in phase one were able to be addressed, disagreements still occurred in theater testing. If the app is going to address a large and diverse risk group, we cannot include niche functionality that may offend some of the target population.

## Introduction

In 2011, gay, bisexual, and other men who have sex with men (MSM) accounted for 62% of new HIV infections in the United States, despite comprising only 2% of the population [1]. To increase identification of new HIV infections and linkage to HIV treatment among MSM, it is recommended that MSM test for HIV at least three to four times per year [2]. However, despite current HIV prevention efforts, most MSM do not test that frequently, with only 20% of MSM testing at least three times per year [3]. This gap identifies a need for new and innovative HIV testing and HIV prevention solutions.

One possible opportunity for innovative HIV prevention is the use of Internet-based interventions and mHealth (the use of mobile phones for medical and public health-supported interventions) [4-9]. mHealth HIV interventions are becoming increasingly popular and include interventions that use mobile text messaging and mobile phone apps [4,10-24]. A study by Muessig et al identified 55 unique mobile apps that address HIV prevention or HIV care, but these apps were not frequently downloaded or highly rated by their users [21]. Even though mobile apps for HIV intervention are a popular platform for developers, it is challenging to encourage app users to download these apps and use them regularly. In order to address this, Muessig et al suggest that prior to building an app, developers should use an iterative data collection process to obtain input from the target audience about app preferences and app evaluation [21].

When disseminating research-based HIV prevention interventions to communities, a disconnect between the research environment and the community can reduce the effectiveness of the intervention and lead to underutilization [25]. Community-based and community-centered approaches towards building research-based interventions can help address the gap between science and practice by ensuring that the interventions are appropriate and driven by the community’s needs and desires [26]. When building mHealth interventions, this means interventions should be developed from community-identified needs [4]. An iterative research process that centers on the community’s needs and preferences (as suggested by Muessig et al) [21] may increase the uptake, adherence, and ultimate effectiveness of mobile apps for HIV prevention. In this study, we outline the use of a community-driven approach to gather data from MSM in order to build an HIV prevention mobile app. This paper describes how the community-driven approach was used to develop an app that was reflective of the reported needs and desires of the community. We outline an iterative app development process in which multiple rounds of interaction with the target community are used to inform and refine the app content and look. We previously published a report of findings from one round of data collection from focus group discussions (FGDs) with MSM addressing men’s preferences for using a mobile HIV prevention app [27]. In the current paper, we build on our previously published work to focus specifically on how multiple rounds of data collection and interaction that constituted the community-driven process allowed us to build a mobile HIV prevention app for MSM that was reflective of their stated needs and desires. The current paper focuses on data from two rounds of data collection, focusing more on the second round of FGDs to illustrate how participant perceptions of the app intervention shifted (or did not shift) after the intervention went through the additional round of app content building. The overall purpose of this paper is to provide a case study to illustrate how an iterative approach to a mobile HIV prevention app can lead to changes in content to appropriately address the preferences of the target community. To achieve this, we present themes that emerged from the data and examine how discussions of these themes shifted at different phases of the study process and how this ultimately lead to the creation of an app that was more likely to be used by the target audience.

## Methods

### Ethics

This study was approved by Emory University’s Institutional Review Board. In this three-phase study, we used FGDs to collect formative data and theater test the app.

### Study Population and Recruitment

Methods for recruitment and a description of the FGDs with MSM during phase one have been previously described [27]. We conducted research with three populations: (1) MSM in Atlanta, Seattle, and rural US regions; (2) HIV testing counselors in Atlanta and Seattle; and (3) key informants including primary care providers as well as key stakeholders at community-based organizations, health departments, and other government agencies at local, county, state, and federal levels. From August to December 2013, we recruited MSM using flyers and Facebook advertisements. Flyers were posted in a variety of venues in Atlanta and Seattle (eg, restaurants, bars, coffee shops, gyms) that MSM are known to frequent. Facebook advertisements targeted men living in Atlanta, Seattle, and rural US regions who reported being interested in men in their profiles. In Atlanta, men recruited through Facebook have been reported to be behaviorally comparable to men recruited through other venues [28]. Rural locations were determined by zip codes and defined as geographical areas with population densities of less than 1000 people per square mile using data from the US Census Bureau [29]. The flyers and advertisements provided a link to an online screening survey through SurveyGizmo (Widgix LLC) to determine eligibility for the study. Participants meeting eligibility criteria for MSM in all three geographical regions were self-identified gay or bisexual men aged 18 years or older who owned or had ever owned a smartphone and had never had a positive HIV test. HIV testing counselors were recruited through flyers sent to organizations, clinics, and health departments in Atlanta and Seattle where HIV testing is performed. HIV testing counselors met eligibility criteria if they were aged 18 years or older, provided HIV testing and counseling services to MSM in Atlanta or Seattle, and owned or had ever owned a smartphone. All participants who completed the online survey and were eligible and interested provided email addresses and phone numbers and were later contacted to participate in an FGD. Some MSM were asked to participate in only one FGD (either in phase one or phase three), and others were asked to participate in both; these participants were randomly selected. Key informants were strategically chosen to provide additional insight into preferences of the users and future collaborations for a smartphone-based HIV prevention app.

### Study Procedures

#### Overview

Other app development studies have used a variety of methods to examine and evaluate mHealth interventions (eg, pre-post test design, interrupted time-series design, randomized controlled testing) [30]. In this study, we applied a simple iterative qualitative approach using FGDs and a beta version of the app to collect preliminary data for building an intervention. The number of FGDs conducted among each population in phases one and three of this study is described in Table 1.

**Table 1:** Outline of focus group discussions.

Focus group discussions		Atlanta	Seattle	Rural	Total
**Phase one**					
	FGDs with MSM	2	2	1	5
	FGDs with counselors	1	1	0	2
Phase one total		3	3	1	7
**Phase three**					
	FGDs with new MSM	1	2	1	4
	FGDS with repeat MSM	1	1	0	2
	FGDs with counselors	1	1	0	2
Phase three total		3	4	1	8
Total		6	7	2	15

#### Phase One: Focus Group Discussions and Key Informant Interviews

For phase one of this study, we conducted FGDs with MSM and HIV testing counselors to get opinions about what should be included in the HIV prevention app, to understand if and how MSM would use the app, and to determine how the app could be incorporated into HIV counseling sessions. We completed four in-person FGDs with MSM (n=28), one online FGD (OFGD) with rural MSM (n=10) [31], two in-person FGDs with HIV testing counselors (n=13), and 14 key informant interviews. The OFGD used a chatroom-based format in Adobe Connect (Adobe Systems Incorporated), a real-time Web-based meeting client. All FGDs lasted approximately 1.5 hours and were conducted by two trained facilitators (one in Atlanta and one in Seattle) who were familiar with the goals of the mobile HIV prevention app.

All FGDs addressed MSM’s general preferences for apps, HIV testing barriers and facilitators, and ways in which an HIV prevention app could address these barriers and facilitators to increase the frequency of HIV testing among MSM. During FGDs, facilitators walked through six images of screenshots to discuss potential functions for a mobile HIV prevention app. Functionality was described by the facilitator, and participants rated each function, providing feedback on why they felt that function would be useful or not useful. Participants also provided suggestions for how to improve each function and the app overall and identified additional functions that should be included.

We also conducted key informant interviews by phone to determine what is feasible and preferable in building the mobile app intervention. Key informants viewed a design document with the same wireframe images of the app. Feedback addressed feasibility of building the app and assessed key informants’ interest in collaborating on building the app.

#### Phase Two: Building a Beta Version of the HIV Prevention App

During phase two, we partnered with Keymind, a division of Axiom Resource Management Inc, to build a beta version of the app. A preliminary analysis of data from phase one was used to build the beta version using a Web-based interactive platform. The mock-up included six major components: (1) navigation aids and pages for personalizing user registration, profile, and privacy and security settings; (2) an interactive HIV testing plan for assessing user testing preferences; (3) a site locator for finding HIV testing facilities; (4) an event tracker for recording sexual encounters, HIV testing dates, and other information relevant to sexual health; (5) frequently asked questions for providing additional HIV prevention tips; and (6) a point system for collecting app interaction credits and donating small denominations of money to organizations focused on HIV and/or lesbian, gay, bisexual, and transgender equity.

#### Phase Three: Theater Testing

After completion of the beta version of the app, we conducted FGDs to theater test the app and solicit opinions on functionality of the app and how it could be used by MSM to improve HIV prevention. We conducted six FGDs with MSM (n=34), two in Atlanta, three in Seattle, and one OFGD with rural MSM. Two of the six FGDs were with MSM who had participated in the first round of FGDs, and four were with newly recruited MSM. We also conducted two in-person theater testing FGDs with newly recruited HIV testing counselors (n=9), one in Atlanta and one in Seattle.

Theater testing was conducted by the same facilitators who conducted the first round of FGDs. For these groups, the facilitator went through the interactive Web-based beta version of the app piece by piece and asked participants to provide feedback on what they liked and did not like about each feature. Facilitators used scenarios to present possibilities for how MSM could use the app. Participants provided feedback on how each function could be used, their willingness to use it, and suggestions for improvement. The purpose of theater testing was to refine the content of the app, determine the best way to present content, and better understand participant attitudes and willingness to use the app.

### Data Analysis

All in-person FGDs were audio-recorded and transcribed verbatim. OFGDs were automatically downloaded to a readable text file. Key informant interviews were not recorded or transcribed, but detailed notes were used to inform the analysis of transcripts from FGDs. Analysis was conducted using MAXQDA version 10 qualitative data analysis software (Verbi GmbH). We conducted a thematic analysis, examining both inductive and deductive themes within the transcripts. After multiple close readings, we created a preliminary codebook of all salient themes. Provisional definitions were given to each code, and four analysts applied each code to a single transcript. The coded transcripts were merged for comparison, and code definitions were revised based on coding disagreements. This process was repeated until a final codebook was created and all four analysts applied codes consistently. Once the final definitions of the codebook were established, analysts consistently applied the codes to all of the fifteen transcripts from both sets of FGDs. Seven of the fifteen transcripts were double-coded with two analysts each coding the same transcript. Eight of the transcripts were coded by one analyst. Double-coded transcripts were merged and codes were reconciled; differences among coders were resolved by consensus. Data were also coded by functionality, with a separate set of inductive codes being applied to all transcripts from phase one and from theater testing. After multiple purposeful and focused readings of coded text, thick descriptions were created for each theme. The descriptions identified common concepts, patterns, and unique ideas expressed in the FGDs. Themes were analyzed separately based on the FGD phase, participant group (MSM or counselors), and location (Atlanta, Seattle, or rural) and were compared and contrasted between groups.

## Results

### Descriptive Statistics

We conducted 15 FGDs with 70 MSM and 22 HIV testing counselors. Nine of the 70 MSM participated in both phases of FGDs. Participant demographics are described in Tables 2 and 3.

### Building on Phase One Results

This three-phase process produced results that enabled researchers and developers to build a detailed design document outlining the functionality of an HIV prevention smartphone app for MSM. Detailed results from FGDs with MSM from phase one have been previously described [27]; however, other data from phase one have not been previously reported. In the first phase of FGDs, MSM described three categories of functions that the app should include: education, interactive engagement, and social networking. MSM also discussed the importance of the tone and privacy of the app. Counselors stated that the app could be a resource during HIV counseling sessions because it could provide educational information, details for risk behavior assessments, previous test dates, etc. Key informants identified potential benefits of the app including benefits for MSM and HIV prevention organizations. Key informants liked that the app could help promote testing and educate MSM about testing, but they also felt that the app should include information about pre-exposure prophylaxis and nonoccupational postexposure prophylaxis and offer more help creating risk reduction plans. Informants also talked about the benefits the app could have for promoting organizations using a locator and potentially allowing MSM to provide feedback on their testing experiences. These informants also identified which functions would be feasible and which would not. For example, we included a wireframe of a function that would validate test results, but community-based organizations and health departments said they do not have the capacity to validate test results on a mobile app. Key informants expressed interest in collaborating and promoting the app.

We used these data from phase one to inform the beta version of the app, resulting in an increased willingness of MSM to use the app during theater testing. Some concerns that were discussed in phase one were addressed in the beta version (eg, disagreements about HIV testing reminders, concerns about app privacy), while others still existed (eg, over functionality, using friendly versus clinical language). We use examples of three app functions (HIV testing reminders, privacy settings, and sex diaries) to explain how the feedback changed throughout the study process. We then examine participant desires for personalizing the app and their willingness to use it.

**Table 2:** MSM participant demographics and HIV testing behaviors.

		Atlanta n=26	Seattle n=26	Rural n=16	Total n=70
Age, years, mean (range)		32.2 (23-53)	40.9 (19-67)	30.8 (19-48)	35.3 (19-67)
**Race, n (%)** ^a^					
	Non-Hispanic white/Caucasian	16 (62)	21 (78)	14 (88)	51 (74)
	Non-Hispanic black/African American	8 (31)	1 (4)	0 (0)	9 (13)
	Other	2 (8)	5 (19)	2 (13)	9 (13)
**Sexual orientation,** **n (%)**					
	Gay/homosexual	24 (92)	26 (93)	14 (88)	64 (91)
	Bisexual	2 (8)	2 (7)	2 (13)	6 (9)
Has had HIV test, n (%)		24 (92)	27 (96)	12 (75)	63 (90)
HIV tests in last 12 months, mean (range)^b^		1.8 (0-4)	1.1 (0-4)	0.7 (0-2)	1.4 (0-4)
**Time since last HIV test, n (%)** ^b^					
	Less than 3 months ago	9 (38)	8 (30)	2 (17)	19 (30)
	3-6 months ago	9 (38)	5 (19)	2 (17)	16 (25)
	6-12 months ago	4 (17)	5 (19)	2 (17)	11 (18)
	More than 1 year ago	2 (8)	4 (15)	5 (42)	11 (18)
	More than 5 years ago	0 (0)	5 (19)	1 (8)	6 (10)
**HIV test site (all that apply), n (%)** ^b^					
	Community-based organization	18 (75)	19 (70.4)	6 (50.0)	43 (68)
	Doctor’s office	19 (79)	20 (74.1)	7 (58.3)	46 (73)
	At home	3 (13)	6 (22.2)	1 (8.3)	10 (16)
	Other	3 (13)	6 (22.2)	1 (8.3)	10 (16)

^a^For reporting of race in Seattle (n=27).

^b^Among MSM who have ever been tested for HIV.

**Table 3:** HIV testing counselor participant demographics.

		Atlanta n=13	Seattle n=9	Total n=22
Age, years, mean (range)		35.9 (23-50)	38.3 (33-50)	37.0 (23-50)
**Race, n (%)** ^a^				
	Non-Hispanic white/ Caucasian	1 (8)	4 (44)	5 (24)
	Non-Hispanic black/ African American	11 (92)	1 (11)	12 (57)
	Other	0 (0)	4 (44)	4 (19)
**Gender, n (%)**				
	Male	6 (50)	6 (67)	12 (57)
	Female	6 (50)	2 (22)	18 (38)
	Gender queer	0 (0)	1 (11)	1 (5)
**Sexual orientation,** **n (%)**				
	Heterosexual	7 (58)	1 (11)	8 (38)
	Gay/homosexual	4 (33)	6 (67)	10 (48)
	Bisexual	1 (8)	1 (11)	2 (10)
	Other	0 (0)	1 (11)	1 (5)
HIV counseling experience, years, mean (range)		2.6 (0.5-9)	3.6 (0.25-12)	3.0 (0.25-12)

^a^Atlanta race demographics are reported on only 12 participants.

### The Impact of HIV Testing Reminders on App Privacy

Privacy and security were salient themes in both rounds of FGDs. In phase one, MSM and counselors in all locations expressed concerns about the privacy of the app. This theme was especially salient when discussing the function of having the app provide reminders for HIV testing:

It’s a matter of privacy with the reminders. I personally don’t care if people see my phone when the notification is going to come up. But some people don’t want that kind of stuff visible.

Atlanta FGD 1, round 1

I still don't like the idea of having to explain this [reminder] if my phone is in a visible area… Push notifications are too visible.

rural OFGD, round 1

MSM in phase one provided suggestions for how to address these concerns about privacy. One suggestion was to use discreet language to refer to an HIV test, for example, making it too vague for anyone else to understand. Participants also offered suggestions for increasing privacy through how the reminder could be delivered (eg, text message, email, app alerts and banners), but participants within groups disagreed on which would be best, concluding that the best solution would be to offer customization for reminders:

I would also agree with what the other comments were around either an email or a text. And I think you should be able to also go in and set your own reminders to say I’d like to get an email every three months to be like hey, you should get tested. I think that would be helpful. I don’t like popups. So I find them to be annoying. But I know for other folks, one of the things I would say is that you have to make sure this app is customizable in a lot of different ways because, just around the table, we all have very different preferences around how we use apps.

Atlanta FGD 2, round 1

I think [reminders are] a good option but overall I think people's preference is so individualized that it would depend on the user the kinds of reminders they prefer.

rural OFGD, round 1

In addition to suggesting customization for the mode of delivery for the reminders, participants also suggested the ability to customize the message itself in order to increase privacy. This would enable participants to choose from a list of messages or write in their own discreet message.

**Figure 1:**
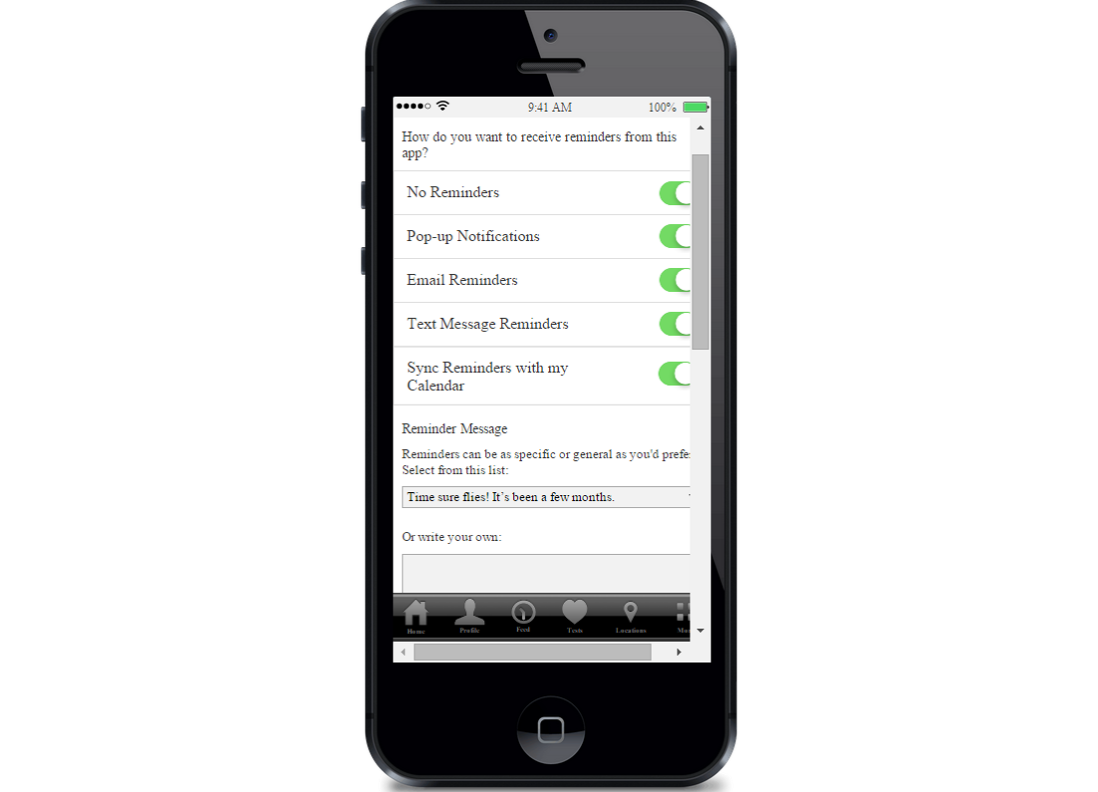
Reminder options.

When we created the beta version of the app, we applied these suggestions and included customized delivery options for reminders, a list of customized messages to choose from, and the option for the app user to write in his own message (Figure 1). We also provided the option of not receiving reminders. In theater testing, this customization solved the concerns about privacy regarding reminders. Participants who were in both sets of FGDs commented on how their issues from the first round of FGDs had been addressed:

I think it’s great. It’s personal, we were worried about this last time, about privacy and personally.… I like the fact that there’s no reminders there…some people don’t want to be reminded. The message is cool. This is exactly what we were worried about like I said last time, and it totally solves that problem.

Atlanta FGD 12, round 2, repeat MSM

MSM who were participating in FGDs for the first time during theater testing expressed that they liked this function and identified which option would be best for them, with different participants choosing different options and describing them as safer in terms of privacy.

### Additional Privacy Settings

While MSM and counselors focused on the reminders when discussing privacy in the first round of FGDs, they also expressed more general concerns regarding the privacy of the app, including the ability for others using the phone to access the app and the security of the data entered into the app. Many participants wondered what would happen with the data and who would have access to it. In theater testing, we added features to secure privacy, including password protection and three privacy setting options: storing the data locally on the phone, storing the data privately in one’s personal cloud, or sharing data anonymously with researchers. Participants really appreciated these options, “Giving people these options will probably cater to everyone” [rural OFGD, round 2]. Nearly all participants stated that they would choose the option of anonymously sharing their data, especially if this would help their community or researchers, help to improve the app, or help app users learn more about patterns of app users in their communities. However, regardless of the reason why a participant would share anonymously, all participants stated that they would only share anonymously if they felt secure in knowing that the data were still protected:

I think it does the most good for society as the larger public health emphasis and all of your data is still protected.

Atlanta FGD 8, round 2, new MSM

I like the idea of having a general idea of how people are behaving locally, but still want to maintain privacy.

rural OFGD, round 2

I would probably be more likely to do [option] three [to share data anonymously], only that I think I would get more out of the app that way.

Seattle FGD 9, round 2, new MSM

Many participants in multiple FGDs advocated so strongly to share data anonymously that they suggested this be the default setting. Alternatively, they suggested forcing app users to address the security settings by having this pop-up on the app when it is first downloaded. Otherwise, participants stated that most app users will simply use the default setting. Being prompted to address the security settings when first downloading the app was also perceived as increasing the overall feeling of security of the app:

If security is the concern it also indicates to them that you thought about security and that this is being addressed upfront rather than like I had to find the security settings

Seattle FGD 13, round 2, repeat MSM

### Sex Diaries and App Tone

In phase one and phase three of this study, MSM disagreed on the tone of the app. This difference occurred between groups and geographical locations, but also within groups. In the first round of FGDs, some participants wanted more fun and friendly language and functionality, identifying that this would make the app more user-friendly and less judgmental. Other participants identified wanting more clinical language and language and functionality that was more authoritative. This was perceived as increasing the credibility and trustworthiness of the app.

**Figure 2:**
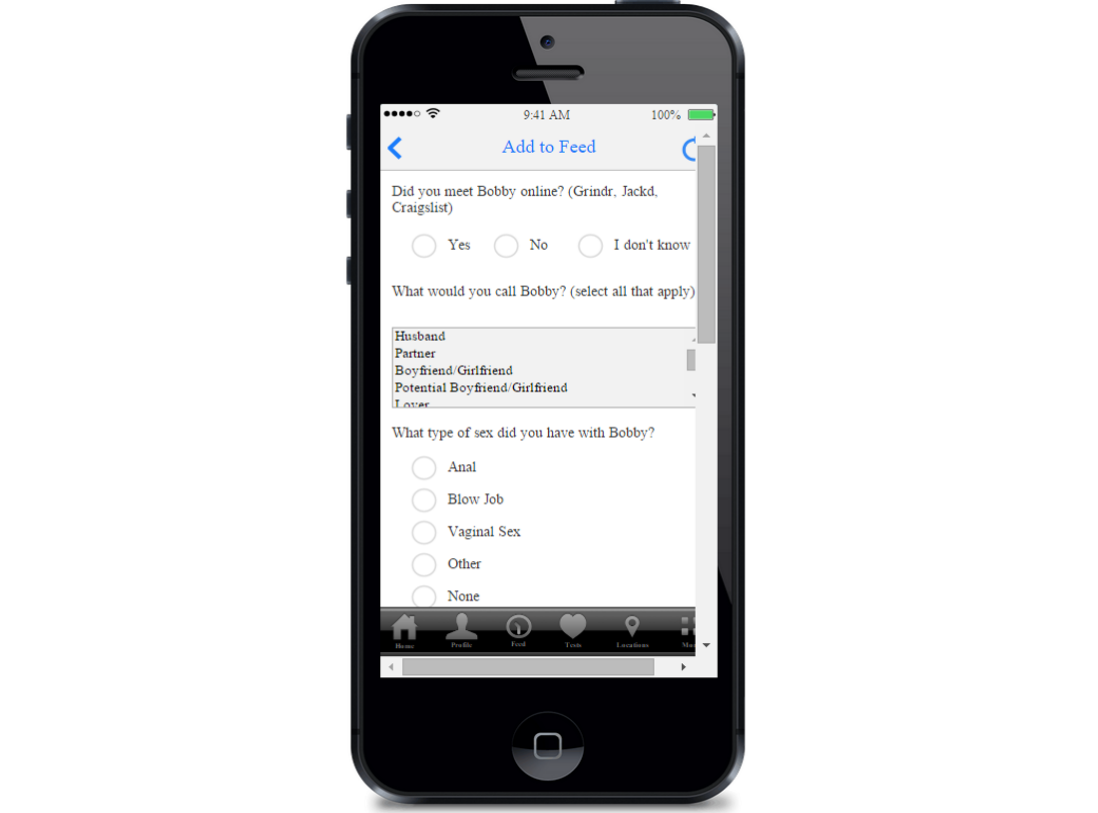
Sex diary.

When creating the beta version of the app, we tried to have content and language that addressed both of these needs. Some functionalities (like descriptions of HIV tests) were straightforward, and while we attempted to use simple, easy-to-understand language for this section, it was not written using sexy language. However, other functionality, like sex diaries in the feed (Figure 2), used more fun and sexy language. The sex diaries were meant to help app users track sex partners and experiences to identify risk behaviors, including condom use and substance use during sex. Some MSM in all three locations strongly expressed support for this feature. Participants liked this function because they felt it was fun and might encourage users to be more engaged with the app, possibly encouraging users to try other pieces of the app:

It does sound like a certain degree of fun, this app overall, like maybe because you’re using this feature of the app, you’ll be more likely to use the other features of the app.

Seattle FGD 9, round 2, new MSM

Participants also found value in this function because they felt it added accountability by being able to keep track of sexual behavior patterns:

I like the idea of tracking your behavior.…I think that that’s important when you’re creating a testing plan to know when and what did you do and when do I need to go get tested. And then, even after I get tested, have I passed the window period or not, so I’ll know if I get tested, do I need to get tested in another couple of weeks, another month or whatever.

Atlanta FGD 12, round 2, repeat MSM

I like it a lot. It makes it feel more like a personal app. Keeping track of things simply adds to the ownership of the whole thing.…People keep a food diary to become healthier. Maybe a sex diary would lead to healthier choices.…And keep one accountable to themselves.

rural OFGD, round 2

Many participants in multiple FGDs liked this feature so much they felt we should center the entire app on this function, use the sex diary for marketing, and call it “My Little Black Book.”

P14: I love the black book idea. I'd market the hell out of this aspect, and use it as tool for HIV testing as the secondary.

Moderator: How do others feel about that?

P18: But, P14, the whole POINT is HIV testing.

P14: I know, but use the fun part of it to get people recording and thinking about their activities and then use that history to encourage testing. …more of a cover on the testing things.

P22: That's true. A Black Book app would be a good angle on it. Maybe Black Book could be incorporated into the title?

P14: Take the clinical aspect out of it.

P19: I feel this is getting a little off-track.

P18: Then again, point. See the list of stuff you've done, and be like, "I should get tested."

P14: Yea… I’m not suggesting dropping the testing, but the diary aspect puts your history in your face, makes you think about it more.

rural OFGD, round 2

Despite the overall positive reaction to this feature in many of the FGDs, not all participants liked this feature. Some, like P19 stated in the OFGD, felt that it took away from the main point of the app.

Even when participants found value in the sex diary function, some participants felt that this function asked for too much from app users and they did not want to put that type of information into their phone:

Are you really going to go to the effort of putting in the dirty details?

rural OFGD, round 2

Sometimes these concerns were related to app user motivation, but participants also identified privacy as a concern regarding this type of sensitive information:

I was the one advocating for blunt use of language but at the same time no one would write that to themselves on the off chance that their mother picked up the phone, but still it’s useful information…even for your own personal diary or your own personal use.

Atlanta FGD 8, round 2, new MSM

It’s just very personal. It’s up to the individual, I mean, I certainly wouldn’t put anything like that on my phone, but somebody that wants to get that detailed and it’s their own personalization, I guess.

Atlanta FGD 12, round 2, repeat MSM

Some participants (especially those in Atlanta) disagreed with this function even further and did not find value in its use. Some of these participants stated that they would delete the app if this were a function on it, even if this function were optional. These participants felt that this function took away from the main point of the app and would encourage MSM to brag to their friends about the number of sex partners they had had:

P1: I’m just kind of uncomfortable now so I don’t think I would put that on my phone…

P2: I would not take this app seriously after seeing this.

P1: I would completely walk away, be done… I don’t see the purpose of writing [about] the sex…Unless you wanted to take this app…

P2: Unless you wanted to make it a game or something.

P3: Well then does it start to defeat its own purpose once you start turning this into like a super fun, how many things can I list out.

P1: Yeah, you start to look at the game and then it’s like…the HIV part becomes, ‘Oh by the way go get tested’ after I’ve done all this.

P2: Knowing that, you know, [Name] blah, blah, blah, has his sex diary on his phone who wants to see his sex diary then he passes his phone around the bar.

Atlanta FGD 8, round 2, new MSM

Some participants who disagreed with this function felt that it would have been improved by a more professional tone:

And this may be just a personal preference for me but I would keep the language… extremely clinical… if I were going to use it I’d want it to be something where I could just put the facts down so that I could have a reminder if I needed it.

Atlanta FGD 8, round 2, new MSM

This disagreement about the sex diary is an example of the tension between making the language and tone of the app overly clinical and over-sexing the language and functionality of the app so that it offends people and deters them from using the app. There was no expressed solution to solve this disagreement, but participants recognized that this tension is a sensitive issue that can make or break an app:

I think there is a fine line between keeping it real and being accessible and trivializing.

Atlanta FGD 8, round 2, new MSM

### Personalization of the App

Participants discussed the importance of personalizing the app. This was evident in the appreciation of customized features like the reminders and the privacy settings. Participants who approved of the sex diaries also stated that they felt it made the app more personal and added to the ownership of the whole thing. In addition to functionality that personalized the app, participants also stated that the language that the app used was personal:

The personal pronouns lend the user towards a sense of ownership of the whole thing.

rural OFGD, round 2

Something that sort of personalizes, like you’ve got MY feed, MY test plan using the word my.

Atlanta FGD 8, round 2, new MSM

Using titles for functions such as “My Test Plan” helped participants to take ownership over these functions and identified that these were functions that could be customized and personalized to fit each app user’s individual needs. Participants liked this type of language so much that they suggested that the title of the app include a personal pronoun to stress the importance of ownership. According to participants, ownership applied to the ability to customize and personalize app functions, but it also contributed to the ownership of one’s sexual health and HIV risk through increased self-responsibility.

### Willingness to Use the App

Overall, participants felt that the app seemed easy to use and the information was easy to digest. Participants appreciated the simple, straightforward language as well as infographics and suggested including more of these. Participants recognized that men most likely to use or need the app are men who are more sexually active, who have concerns about their HIV risk, or who do not already have a HIV testing plan established.

One of the biggest challenges identified by participants in getting men to use the app is maintaining interest and motivation to use the app:

It seems like one of those apps that you download and you play it for about ten minutes after you downloaded it and then just kind of sat there on your phone until you get the notification.

Atlanta FGD 8, round 2, new MSM

Even though some participants expressed that this might not be an app that they would use constantly, participants did see the importance for having this app available when they really needed it:

But I think too if you do have an accident or you have engaged in high risk behavior…and you’re scared and you don’t have a plan in place you might turn to this out of curiosity to help build a plan. It would be a good anonymous way to…create a plan and find out as much as you want to find out too. But…I wouldn’t play on it every day but again we’ve all had.

Atlanta FGD 8, round 2, new MSM

Participants also offered suggestions for what could help motivate MSM to use the app more regularly. One idea was to incentivize app use. In the beta version, we included incentives in the form of reward points that would contribute to donations for organizations, but participants stated that there should also be incentives that benefit the app user directly:

I was trying to think of what would get me or get some people I know to do it, and it might be like, I was wondering if you could do, you know, fifty points and we’ll send you a pack of condoms, or a hundred points and you get no cover charge at this club.…I think [the reward points are a] great thing, I don’t know if it’s going to motivate as many people as something that’s actually for them.

Seattle FGD 10, round 2, new MSM

In addition to incentivizing, many participants in multiple FGDs discussed app promotion and advertising as an important way to get users to download the app.

## Discussion

### Principal Findings

These findings highlight how using an interactive and community-centered process to collect data on app preferences is fundamental when building a mobile HIV prevention app. Many of the concerns and problems that were voiced in the first round of FGDs were addressed in the beta version of the app with increased acceptability noted in phase three, especially regarding concerns about privacy. Through this process, we learned about the needs and desires that MSM have for a mobile HIV prevention app and gained insight on what would motivate men to download and use the app.

Through the testing, we learned that if this app-based intervention is going to address a large and diverse risk group, we cannot include niche functionality that may offend some of the target population. Even though some participants loved the sex diaries, others said that they would not use the app at all if it was included, even as an optional function. This app is meant to cater to the larger MSM population, so it needs to include more general functionality that everyone agrees is useful while also being customizable so that each app user can have a personalized experience. In the process of building this app, we learned that personalization and customization can improve many components of the app, especially when there are personalized settings to address different user security needs. This personalization along with interactive functionality allows for the app user to take ownership over the app, making HIV testing plans and other features more catered to the app users’ specific needs. According to participants, this act of taking ownership over one’s sexual health within the app may also assist men to take ownership of their HIV risk management in other aspects of their lives.

This concept of personalization and ownership is aligned with Bandura’s social cognitive theory of self-regulation, which states that self-regulation occurs through self-monitoring, judging one’s behaviors in relation to personal and societal standards, and reacting to these judgments [32]. Participants who appreciated the sex diaries expressed the benefits of self-regulation and increased accountability. Participants also recognized other areas of the app that encouraged this type of accountability, such as the use of personal pronouns (eg, My Test Plan). These findings support current recommendations that mHealth interventions should be guided by existing theories of behavior change [33]. HIV prevention apps may benefit from applying this cognitive theory of self-regulation through increased personalization, accountability, and ownership of app functionality.

### Limitations

Although recruitment by race is reflective of the larger geographical demographics [34] with more black MSM participating in Atlanta, we do not have enough racial variation to make any conclusions based on race. Participants represented an older population of MSM with an overall mean age of 35. We do not know how the results would have differed if they were more reflective of the attitudes of younger MSM, and given the increase in HIV among young MSM, this represents a knowledge gap in the current study. Participants in Seattle also had a higher mean age than participants in Atlanta and we do not know if this contributed to any differences between geographical groups. In addition, recruitment of men who identify as gay or bisexual and who identify as seeking other men on Facebook may not represent MSM in general. Including only MSM who own smartphones may also not be representative of MSM in general; however, targeting MSM who owned smartphones allowed us to include participants who would be most likely to use the app. Since these are qualitative findings, we are not trying to generalize study results or quantify differences between groups. However, we were able to understand app preferences using populations in two different US cities as well as rural regions, where population demographics, culture, and HIV prevention efforts vary. OFGDs with rural men were limited to the online environment. All participants needed to have access to a computer with Internet. Furthermore, the facilitator did not have nonverbal cues to assist with probing questions. Still, the OFGDs were useful in capturing insight from a population that we would have otherwise not been able to include in this study. Overall, this study addressed perspectives and attitudes on willingness to use an HIV prevention app and to determine the best approach for functionality and content of the app; however, this study did not address usability testing. While usability testing is important to ensure that the target population will use the app, since this paper describes an early stage of app development, the aim of the FGDs was to determine preferences for content and functionality rather than design and interface. Future testing of the app will address other factors that influence app usability.

### Conclusions

Despite these limitations, this study reflects the need for a community-driven approach that includes multiple rounds of data collection and theater testing when developing apps or other mHealth interventions for HIV prevention. Building an HIV prevention app is expensive and requires time and resources. To maximize app uptake and usage, it makes sense to build the best app possible, and the definition of best app should be defined by the community it aims to serve. Through this process, we learned how to be inclusive of the larger MSM population without marginalizing some app users. We also learned how to personalize the app so users take ownership and feel comfortable with its security. This community-driven process increased an overall willingness to use the app and provided important insight into how to build an HIV prevention app that MSM want to use.

## Abbreviations

FGD: focus group discussion
MSM: men who have sex with men
OFGD: online focus group discussion
